# Filtering procedures for untargeted LC-MS metabolomics data

**DOI:** 10.1186/s12859-019-2871-9

**Published:** 2019-06-14

**Authors:** Courtney Schiffman, Lauren Petrick, Kelsi Perttula, Yukiko Yano, Henrik Carlsson, Todd Whitehead, Catherine Metayer, Josie Hayes, Stephen Rappaport, Sandrine Dudoit

**Affiliations:** 1 Division of Biostatistics, UC Berkeley, Berkeley, 94720 USA; 2 The Senator Frank R. Lautenberg Environmental Health Sciences Laboratory, Department of Environmental Medicine and Public Health, Icahn School of Medicine at Mount Sinai, New York, USA; 3 Department of Statistics, UC Berkeley, Berkeley, 94720 USA; 4 Division of Environmental Health Sciences, UC Berkeley, Berkeley, 94720 USA; 5 Division of Epidemiology, UC Berkeley, Berkeley, 94720 USA; 6 Center for Integrative Research on Childhood Leukemia and the Environment, UC Berkeley, Berkeley, 94720 USA

**Keywords:** Metabolomics, Filtering, Preprocessing, Data-adaptive

## Abstract

**Background:**

Untargeted metabolomics datasets contain large proportions of uninformative features that can impede subsequent statistical analysis such as biomarker discovery and metabolic pathway analysis. Thus, there is a need for versatile and data-adaptive methods for filtering data prior to investigating the underlying biological phenomena. Here, we propose a data-adaptive pipeline for filtering metabolomics data that are generated by liquid chromatography-mass spectrometry (LC-MS) platforms. Our data-adaptive pipeline includes novel methods for filtering features based on blank samples, proportions of missing values, and estimated intra-class correlation coefficients.

**Results:**

Using metabolomics datasets that were generated in our laboratory from samples of human blood, as well as two public LC-MS datasets, we compared our data-adaptive filtering method with traditional methods that rely on non-method specific thresholds. The data-adaptive approach outperformed traditional approaches in terms of removing noisy features and retaining high quality, biologically informative ones. The R code for running the data-adaptive filtering method is provided at https://github.com/courtneyschiffman/Metabolomics-Filtering.

**Conclusions:**

Our proposed data-adaptive filtering pipeline is intuitive and effectively removes uninformative features from untargeted metabolomics datasets. It is particularly relevant for interrogation of biological phenomena in data derived from complex matrices associated with biospecimens.

**Electronic supplementary material:**

The online version of this article (10.1186/s12859-019-2871-9) contains supplementary material, which is available to authorized users.

## Background

Metabolomics represents the small-molecule phenotype that can be objectively and quantitatively measured in biofluids such as blood serum/plasma, urine, saliva, or tissue/cellular extracts [1–4]. Untargeted metabolomics studies allow researchers to characterize the totality of small molecules in a set of biospecimens and thereby discover metabolites that discriminate across phenotypes [1, 3, 5]. Among the techniques employed for untargeted metabolomics, liquid chromatography-high-resolution mass spectrometry (LC-HRMS) has become the analytical tool of choice due to its high sensitivity, simple sample preparation, and broad coverage of small molecules [2, 6]. However, many of the thousands of features detected by untargeted metabolomics are not biologically interesting because they represent background signals from sample processing or multiple signals arising from the same analyte (adducts, isotopes, in-source fragmentation) [7]. Furthermore, feature detection and integration with software such as *XCMS* [8] is imperfect, in that noise can erroneously be identified as a peak group, the domain of integration can be incorrect, etc. Thus, large metabolomics datasets can contain thousands of falsely identified features or features with imperfect integration (e.g., incorrect integration regions and missing values).

Inadequate feature filtering can affect subsequent statistical analysis. For example, if high quality features are erroneously filtered, they will not be considered as candidate biomarkers in univariate tests of significance for association with biological factors of interest or in metabolic pathway analysis. Furthermore, if one performs univariate tests of significance and ranks features based on *p*-values, biologically meaningful features could be lost in an abundance of noise without adequate feature filtering. Failure to filter noise could also result in false positives when assessing the significance of metabolic pathways with software such as *Mummichog*, which relies on sampling features from the entire dataset to create null distributions of pathway statistics [9].

Therefore, untargeted metabolomic data require a set of filtering methods to remove noise prior to investigating the biological phenomena of interest. Data normalization has received a lot of recent attention in untargeted metabolomics [10–14]. Feature filtering, however, remains a fairly automated, indelicate, and brief step in the preprocessing of untargeted metabolomic data. Many studies rely on valuable preprocessing pipelines offered from programs like *Metaboanalyst* and *Workflow4Metabolomics* to process their raw data. Such programs have greatly advanced the field of untargeted metabolomics and have improved data pre-processing and analysis and replication of results. However, many users of these programs rely on the provided, default cutoffs for feature filtering, and do not attempt to identify more appropriate, data-specific filtering cutoffs.

For example, *MetaboAnalyst* allows users to filter features based on mean/median value across samples, as wells as variability across biological samples and quality control (QC) samples. While these are indeed useful filtering metrics, most users do not determine the filtering thresholds appropriate for their specific data. *Metaboanalyst* suggests removing the lowest *k* percent of features based on the size of the dataset (e.g., lowest 40% of features for a dataset with more than one thousand features based on mean/median abundance across samples), and a relative standard deviation (RSD, the same as a coefficient of variation or CV) cutoff of 25% for LC-MS data [12]. While these are helpful guidelines for selecting cutoffs, users often fail to investigate whether they are appropriate for their data. Similarly, *Workflow4Metabolomics*, for good reasons, allows users to filter features based on variability across replicates and sample mean vs. blank mean ratios, but many users continue to rely on default or commonly used cutoffs. Here we offer researchers alternatives to default filtering cutoffs that may be more appropriate for their datasets.

**Fig. 1:**
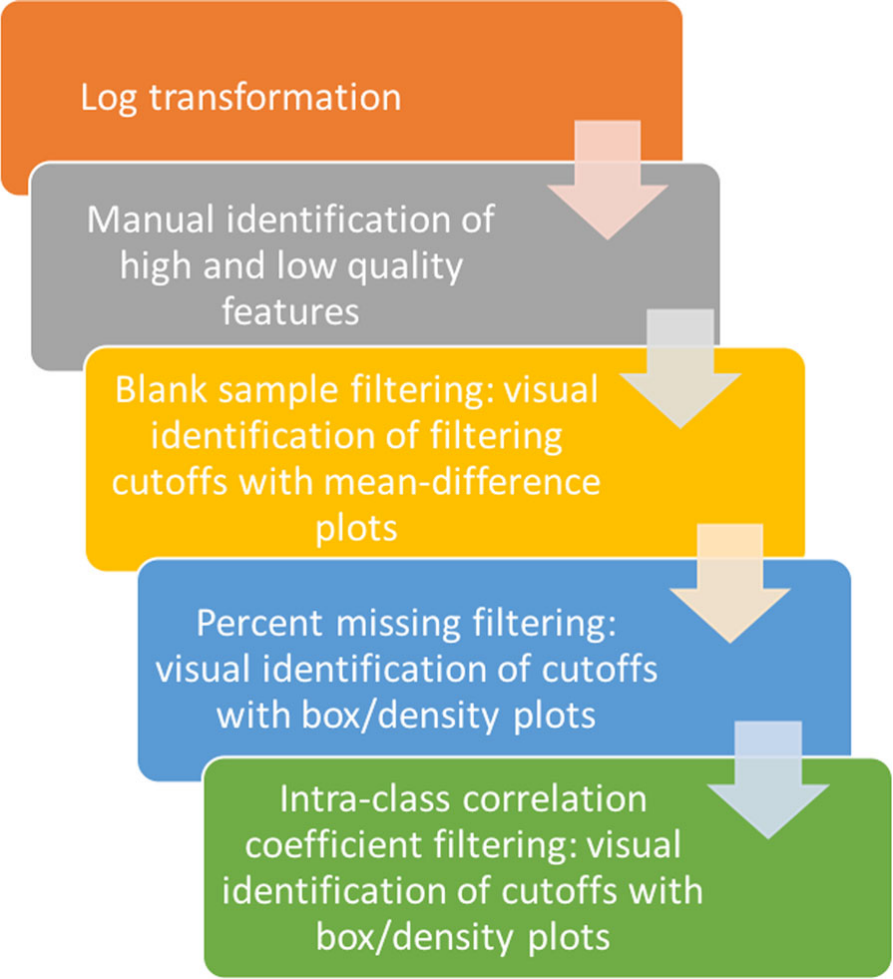
Filtering flowchart. Flowchart of a data-adaptive filtering pipeline for untargeted metabolomics data. The pipeline is data-adaptive because the filtering cutoffs used are specific to the filtering needs of the data at hand

We argue that filtering methods should be data-adaptive. A data-adaptive pipeline is one which tailors filtering to the specific characteristics of a given dataset, rather than using predefined methods. In what follows, we present a series of steps (Fig. 1) representing a data-adaptive pipeline for filtering untargeted metabolomics data prior to discovering metabolites and metabolic pathways of interest. Our data-adaptive filtering approach contains novel methods for removing features based on blank sample abundances, proportions of missing values, and estimated intra-class correlation coefficients (ICC). To create data-dependent thresholds for the above three feature characteristics, we propose visualizing the differences in the characteristics between known high and low quality features. By examining such differences for each dataset, one can minimize noise without compromising the underlying biological signal. Once this is done for several datasets generated from a given laboratory, the determined filtering cutoffs may be appropriate to other similar datasets. Properly filtered untargeted metabolomic data can then be used as input into valuable processing pipelines such as *MetaboAnalyst* and *Workflow4Metabolomics* for further preprocessing and data normalization. We compare our data-adaptive filtering method to common filtering methods using two untargeted LC-HRMS datasets that were generated in our laboratory (see also Additional file 1) and two public LC-MS datasets obtained on a different analytical platform. To compare the methods, we identified hundreds of high and low quality peaks in each dataset. We then showed how our data-adaptive pipeline surpasses workflows that use default cutoffs to remove low quality features while retaining high quality features.

## Methods

### Visualizing high and low quality features

**Fig. 2:**
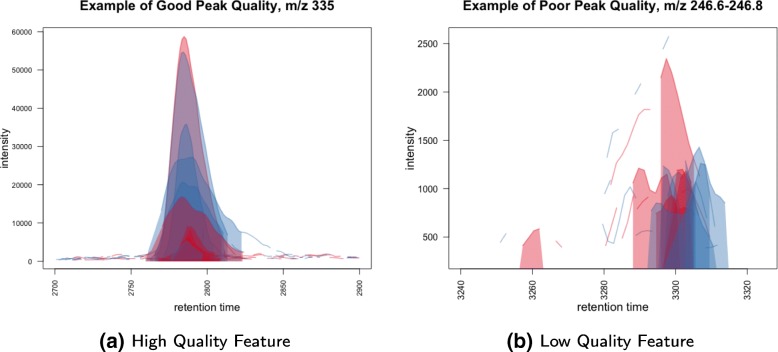
Example of a high and low quality peak group. The peak groups depicted here are examples of features with (**a**) high and (**b**) low quality peak morphology and integration. We followed the *XCMS* R package vignette to process the example LC-MS dataset provided in the package [8]

When working with untargeted LC-MS data, visualization of extracted ion chromatograms (EIC) of features can be used to optimize peak detection, peak quantification, and biomarker discovery [8, 15, 16]. We propose randomly sampling several hundred EICs after peak detection and quantification to visualize peak morphology and integration. The EICs can then be classified by the user as “high” or “low” quality (see Fig. 2). A high quality peak has good morphology (e.g., is bell-shaped, although this is not a necessary condition), the correct region of integration across all samples, and proper retention time alignment. Such visualization is made easy with plotting functions from peak detection software such as the ’highlightChromPeaks’ function within *XCMS* [8]. In almost all cases, we find the distinction between high and low quality peaks to be clear, but when peaks are ambiguous we make the conservative choice to classify them as low quality. Once features are classified as high or low quality, their characteristics across samples such as average blank and biological sample abundance, percent missing, and ICC can be compared and used to perform feature filtering. While classification of high and low quality peaks is a time intensive step, we have found that visualization and inspection of hundreds of features takes between 1–2 h and greatly improves the ability to uncover biological variability in the data. Moreover, after feature visualization, executing the remaining steps of the filtering pipeline requires no more than 1 h.

### Data-adaptive feature filtering

#### Example datasets

To help present and visualize our data-adaptive feature filtering methods, we introduce an untargeted LC-HRMS dataset generated in our laboratory on a platform consisting of an Agilent 1100 series LC coupled to an Agilent 6550 QToF mass spectrometer. The dataset contains the metabolomes of 36 serum samples from incident colorectal cancer (CRC) case-control pairs as described in [16, 17]. Over 21,000 features were detected in the 36 serum samples that were analyzed in one batch [16, 17]. We randomly sampled over 900 features from the dataset and classified these as “high” or “low” quality according to their peak morphology and integration quality. To demonstrate the performance of our data-adaptive pipeline, we split the known high and low quality features into a training set (60%) and a test set (40%). Features in the training set were used to visualize appropriate, data-dependent cutoffs, whereas features in the test set were used to evaluate the effectiveness of the selected cutoffs. At each stage of the data-adaptive filtering, we compared our method to more traditional filtering methods by examining the proportions of high and low quality features in the test set that were removed. An application of the data-adaptive pipeline to another untargeted LC-HRMS metabolomics dataset generated in our laboratory can be found in Additional file 1. This additional dataset represents the metabolomes of 4.7-mm punches from archived neonatal blood spots (NBS) of 309 incident case subjects that were obtained for the California Childhood Leukemia Study [15, 18]. For the sake of clarity, we do not include results for this second dataset in the main text, and the results can be found instead in Additional file 1.

We also visualized and classified over 200 features in each of two public LC-MS datasets. One of the public datasets was generated on a platform consisting of an Accela liquid chromatographic system (Thermo Fisher Scientific, Villebon-sur-Yvette, France) coupled to an LTQ-Orbitrap Discovery (Thermo Fisher Scientific, Villebon-sur-Yvette, France). This dataset contains the metabolomes of 189 human urine samples analyzed in negative mode. We took a subset of 45 of the urine samples in the first batch, along with 14 pooled QC samples and 5 blank samples. We processed this dataset using the original *xcms* functions and parameters used by the authors (W4M00002_Sacurine-comprehensive) [10, 19]. The second public dataset was generated on a platform consisting of an Accela II HPLC system (Thermo Fisher Scientific, Bremen, Germany) coupled to an Exactive Orbitrap mass spectrometer (Thermo Fisher Scientific) [20]. This dataset contains the metabolomes of epithelial cell lines treated with low and high concentrations of chloroacetaldehyde. We used all 27 cell line samples in negative mode treated with low concentrations, as well as 6 pooled QC and 11 blank samples. The original work did not use *xcms* to process the raw data, so we used the R package *IPO* to determine the *xcms* parameters [21].

#### Filtering features based on blank samples

Blank control samples, which are obtained from the solvents and media used to prepare biological samples, can help to pinpoint background features that contribute to technical variation [2, 3, 10, 22, 23]. A common filtering method is to use a fold-change (biological signal/blank signal) cutoff to remove features that are not sufficiently abundant in biological samples [3, 10, 12]. Rarely does the user examine the data to determine a suitable cutoff. We employ a data-adaptive procedure that takes into account the mean abundance of features in blank and biological samples, the difference between mean abundances in blank and biological samples, and the number of blank samples in which each feature is detected. Our method then assigns cutoffs according to the background noise and average level of abundance. If the dataset contains several batches, filtering is performed batch-wise.

**Fig. 3:**
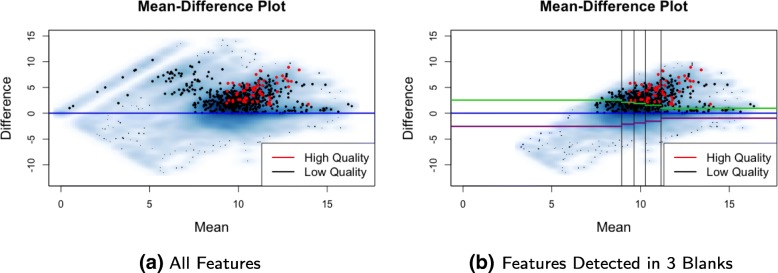
MD-plot for the CRC dataset. **a** Four clusters of features can be identified in the MD-plot, corresponding to features detected in zero, one, two and all of the three blank samples. For this dataset, all high quality features (in red) are in the cluster of features with the highest average abundances that are detected in all three blank samples. **b** Because all of the high quality features in the training set are detected in all three blank samples, we remove any features detected in less than three blank samples. We filter features detected in all blank samples (shown here) by using the distribution of the known noise below the zero difference line (in blue) to estimate the noise above the zero difference line. We use the absolute value (green lines) of the lower quartile of the negative differences (purple lines) within each partition (20th, 40th, 60th, and 80th percentiles) as filtering cutoffs. Any features above the green lines are retained

We use a mean-difference plot (MD-plot) to visualize the relationship between feature abundances in the blank and biological samples and assess background noise (Fig. 3). Abundances are log transformed prior to all data pre-processing and visualization. The mean log abundances of each feature across biological and blank samples are then calculated and the average of and difference between these two means are then plotted on the x- and y-axes, respectively. The horizontal zero-difference line (blue lines in Fig. 3) represents the cutoff between features having higher mean abundances in the blank samples and those having higher mean abundances in the biological samples. If there are *n* blank samples in a batch, then *n*+1 clusters of features will typically be visually identifiable in the MD-plot, where cluster *i*=0,…,*n* is composed of features that are detected in *i* blank samples. For example, because three blank samples per batch were used in the example dataset, four clusters are identifiable in Fig. 3a. Similar clusters can be identified in all datasets generated from our laboratory (See Additional file 1: Figure S1) and in the public datasets. Filtering is then performed separately for each cluster. If a cluster contains no high quality features, as is often the case with clusters that contain lower abundance features, that cluster can be removed entirely.

The cluster corresponding to features detected in all *n* blank samples tends to have the highest number of features (around 95% of the total number of features), features with higher average abundances, and the highest number of high quality features. Therefore, careful, data-dependent filtering of this cluster is crucial for the success of subsequent analyses. This cluster also has a non-uniform distribution of mean feature abundances (Fig. 3b). This cluster is thus partitioned based on quantiles (20th, 40th, 60th, and 80th percentiles) of the empirical distribution of mean abundances (x-axis). This ensures that each partition has the same number of features and that the features are uniformly distributed throughout the dynamic range. Within each partition, the empirical distribution of abundances below the zero-difference line is used to estimate the technical variation above that line. The absolute value (green lines in Fig. 3b) of an appropriately identified percentile of the negative mean differences (purple lines in Fig. 3b) is used as a cutoff to remove uninformative features. Users may identify appropriate percentiles of the negative mean differences (purple lines) based on how many high quality features would be removed if the absolute values of those percentiles (green lines) were used as cutoffs. We find percentiles between the lower quartile and median to be appropriate for this cluster of features, because they remove as many low quality features as possible without removing high quality ones. Feature filtering in the remaining clusters can be performed in a similar manner, but without the need to partition features based on average abundance.

**Fig. 4:**
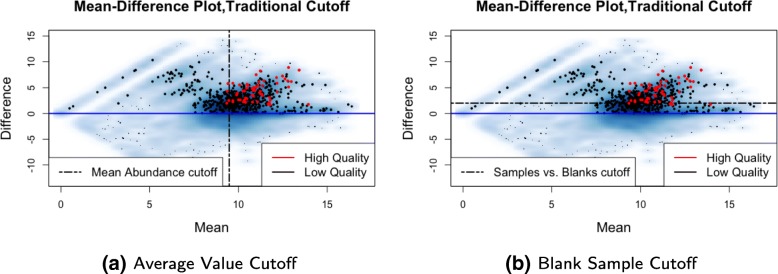
Two traditional filtering cutoffs. **a** For datasets with more than one thousand features, *MetaboAnalyst* recommends removing the lowest 40% of features according to average abundance [12]. The vertical line shown here is an example of such a cutoff, with all features to the left of the vertical line being removed. With this cutoff, many features with higher average abundance in blank samples (below the blue line) are still retained. **b** Some filtering methods use a pre-specified cutoff for the ratio or difference between average abundance in blank versus biological samples [10]. The black horizontal line shown here represents such a cutoff. All features with a difference in average abundance between biological and blank samples less than two (below the black line) would be removed. If such a traditional filtering approach is to be used, the MD-plot can, at the very least, help users to identify a more appropriate, less arbitrary cutoff for their dataset that strikes a better balance between removing low quality features and retaining high quality ones

Using MD-plots to filter features allows for the simultaneous filtering of features by both the difference in abundance in blank and biological samples (y-axis) and average abundance (x-axis). Average abundance of features across biological samples is a commonly used filtering characteristic, but the filtering is often done using pre-specified cutoffs (e.g., lowest forty percent for datasets with more than one thousand features) (Fig. 4a) [10, 12]. Although we advocate for the filtering approach described previously, if users prefer to filter by just average abundance, the MD-plot allows for easy visualization of a data-dependent cutoff that removes as many low quality features as possible without removing high quality ones. The same can be said for identifying a data-adaptive fold-change (biological signal/blank signal) cutoff, rather than using default cutoffs provided in preprocessing workflows (Fig. 4b) [10]. While we recognize that the background signal can modify the biological signal (e.g., via ion suppression), we do not consider this source of variability.

### Filtering features by percent missing

**Fig. 5:**
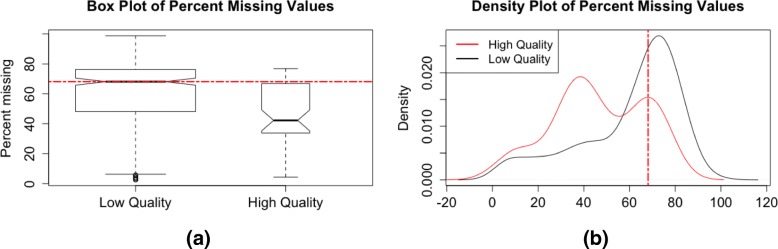
Distributions of percent missing for high and how quality peaks in the training set. Using (**a**) box plots and (**b**) density plots of the percent missing values for high and low quality features in the training set, we chose a filtering cutoff of 68% missing, the median value of percent missing for the low quality features in the training set. The plots help to visualize and compare the modes and percentiles of the two distributions (for low and high quality features). The median of the distribution for low quality features is greater than the mode and even 90th percentile of the distribution for high quality features in the training set, making it an appropriate cutoff

As mentioned above, low-abundance metabolomic features tend to have a high proportion of undetected values across samples. In addition, when using software such as *XCMS* for peak detection and quantification, peaks can be missed by the first round of peak detection and integration. Functions such as ’fillChromPeaks’ in *XCMS* are often used to integrate signals for samples for which no chromatographic peak was initially detected [8, 12]. Low quality peaks also tend to have higher proportions of missing values after initial peak identification and integration (Fig. 5 and Additional file 1: Figure S2).

To determine the appropriate filtering cutoff for percent missing, we create side-by-side box plots of percent missing values for the high and low quality features classified by visualization of EICs (Fig. 5a). The box plots help to compare the percentiles of the distributions of percent missing values for the high and low quality features, and to select an appropriate cutoff based on these percentiles. Density plots of percent missing values can also be used to visualize the modes and percentiles of the distributions for high and low quality features (Fig. 5b), and cutoffs can be determined based on these distributional properties. For example, appropriate cutoffs would be those that discriminate between the modes of the two distributions, that remove long tails of distributions of low quality features, that correspond to extreme percentiles of one distribution but intermediate percentiles of another, etc. To ensure that we do not remove features that are differentially missing between biological groups of interest (e.g., mostly missing in cases but not controls), we perform a Fisher exact test for each feature, comparing the number of missing and non-missing values against the biological groups of interest. A small *p*-value for a given feature would indicate that there is a significant dependence between the phenotype of interest and missing values. Features with a percent missing below the identified threshold or with a Fisher exact *p*-value less than some threshold (we recommend a small value such as the one hundredth percentile of the *p*-value distribution) are retained. This test of association between the phenotype of interest and missing values can easily be extended to studies where the biological factor of interest is a multilevel categorical variable or a continuous variable by using, for example, a Chi-Square test or a Wilcoxon rank-sum test, respectively.

### Filtering features by ICC

**Fig. 6:**
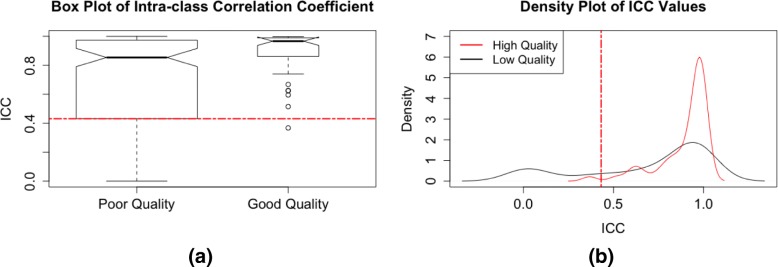
Distributions of estimated ICC values for high and low quality peaks in the training set for the CRC data. We use (**a**) box plots and (**b**) density plots to visualize the modes and percentiles of the distributions of the ICC values for high and low quality features in the training set, and choose a filtering cutoff of 0.43. The high quality features have a mode close to one, and the distribution of the low quality features has a larger left tail, suggesting that a cutoff to the left of the mode of the high quality features would be appropriate

**Fig. 7:**
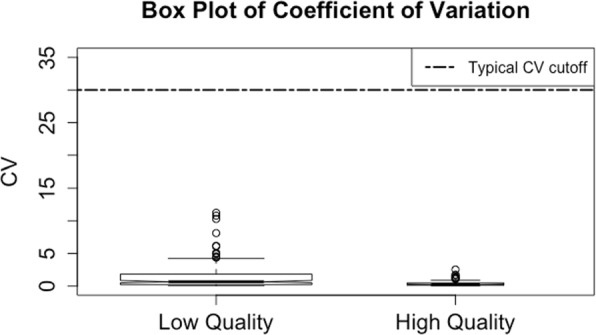
Box plot of CV values in the CRC dataset. A typical CV filtering cutoff is 30% (horizontal black line) [10]. For the CRC dataset, this cutoff does not remove any of the low quality features

High quality and informative features have relatively high variability across subjects (biological samples) and low variability across replicate samples [10, 12] (Fig. 6 and Additional file 1: Figure S3). Typically, the coefficient of variation (CV) is calculated across pooled QC samples for each feature and those with a CV above a predetermined cutoff (e.g., 20–30%) are removed [1, 2, 10, 12, 22]. However, we find that the CV is often a poor predictor of feature quality (Fig. 7 and Additional file 1: Figure S4) because it only assesses variability across technical replicates, without considering biologically meaningful variability across subjects. Instead, we propose examining the proportion of between-subject variation to total variation, otherwise known as the intra-class correlation coefficient (ICC) [24], as a characteristic for filtering. Since the ICC simultaneously considers both technical and biological variability, a large ICC for a given feature indicates that much of the total variation is due to biological variability regardless of the magnitude of the CV.

Our method for estimation of the ICC employs the following random effects model:

1 $$\begin{array}{@{}rcl@{}} Y_{i,j} = \mu_{j} + b_{i,j} + \epsilon_{i,j,k}, \end{array} $$

where *Y*_*i,j*_ is the abundance of feature *j* in subject *i*, *μ*_*j*_ is the overall mean abundance of feature *j*, *b*_*i,j*_ is a random effect for feature *j* in subject *i*, and *ε*_*i,j*,*k*_ is a random error for replicate measurement *k* for feature *j* in subject *i*. The ICC is estimated by taking the ratio of the estimated variance of *b*_*i,j*_ (between-subject variance) to the estimated variance of *b*_*i,j*_+*ε*_*i,j*,*k*_ (total variance). If replicate specimens or LC-MS injections are analyzed for each subject, then application of Eq. 1 is straightforward. However, since metabolomics data are often collected with single measurements of each biospecimen and employ repeated measurements of pooled QC samples to estimate precision, then Eq. 1 can be fit by treating the pooled QC samples as repeated measures from a ’pseudo-subject’. As with percent missing, density plots and box plots of the estimated ICC values for high and low quality features can be compared to determine a data-specific filtering cutoff (Fig. 6). Again, we look to the modes and percentiles of the distributions of the high and low quality features to select an appropriate cutoff that strikes a balance between removing low quality peaks and retaining high quality ones. If multiple batches are involved, the final feature list represents the intersection of features from all batches.

## Results

**Fig. 8:**
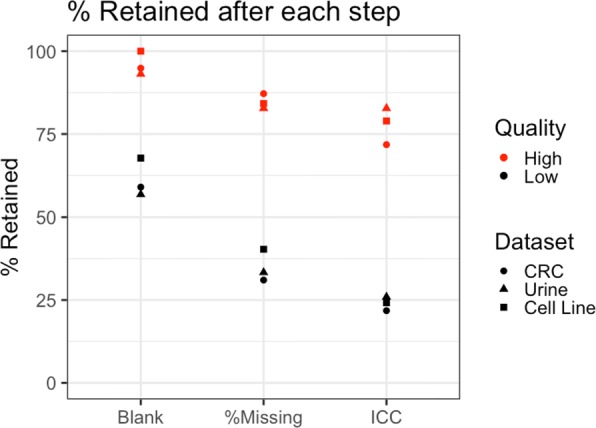
Percent of high and low quality features in the test set remaining after each filtering step. Each step of the proposed data-adaptive filtering pipeline considerably reduces the number of remaining low quality features in the test set. The desired trade-off between removing low and high quality features can be obtained by adjusting the stringency of the cutoffs at each step. For the CRC dataset, 76% of the low quality features and 28% of the high quality features in the test set were removed. For the urine dataset, 74% of the low quality features and 17% of the high quality features in the test set were removed. For the cell line dataset, 76% of the low quality features and 21% of the high quality features in the test set were removed

The MD-plot for the CRC dataset shows that all high quality features in the training set are in the same cluster corresponding to features detected in all three blank samples (Fig. 3). Because features in this cluster have higher average abundances and lower percent missing than those in the other three clusters, it is not surprising that this cluster is comprised of many high quality peaks. We therefore remove features in the other three clusters for this dataset, and focus on the data-adaptive filtering of the cluster containing the high quality features (Fig. 3b). We use the lower-quartile of noisy features below the zero difference line to estimate the noise above the zero difference line because this cutoff removes a considerable number of low quality features without removing many of the high quality features (Fig. 3b). This threshold in the training set was then applied to the test set. In fact, this filtering step removed 68% of the 21,000 features, and 41% of the identified low quality features in the test set (Fig. 8). Almost all (95%) of the high quality features in the test set were retained (Fig. 8). A common approach to filtering would be to remove features based on their mean abundance, such as removing the lowest 40% [12]. If this threshold were used to filter the CRC dataset, only 31% of the identified low quality features in the test set would be removed, and many remaining features would have higher average abundances in the blank samples (Fig. 4). Another traditional approach is to arbitrarily select a cutoff (2–5) for the ratio between average biological and blank sample abundances. A similar cutoff applied to the CRC dataset (a cutoff of two for the difference between average log abundances in biological and blank samples) would remove only 36% of the low quality features and 10% of the high quality features in the test set, and would fail to remove many of the low quality features in the clusters that are removed by our data-adaptive filtering. Utilizing blank samples in filtering certainly helps to reduce the number of low quality features. Furthermore, utilizing data visualization helps to ensure that filtering is done appropriately, i.e. that an appropriate balance is struck between removing low quality features and retaining high quality ones.

The next step in the data-adaptive filtering is to visualize differences in percent missing among the remaining high and low quality features (Fig. 5). Using the information on distribution modes and percentiles provided by box plots and density plots of the data in the training set, we chose to remove features with more than 68% missing values (median of percent missing for low quality features). This threshold was then applied to the test set. When a Fisher exact test was used for each feature to detect significant associations between missing values and the biological factor of interest (CRC), 68 features had *p*-values less than 0.027 (the one hundredth percentile of the *p*-values) and were retained regardless of their percent missing values. Combining these two filtering criteria removed 47% of the remaining low quality features and only 11% of the remaining high quality features in the test set (Fig. 8).

We used the 12 QC samples from the CRC dataset to calculate ICC values for each of the remaining features. Using the information provided by the density and box plots, we chose to remove features with ICC values less than 0.43 (the lower hinge of the box plot for low quality features in the training set) (Fig. 6). This threshold was then applied to the test and removed 23% of the remaining low quality features and only 15% of the remaining high quality ones (Fig. 8). Compare this to using CV values to perform filtering, where a typical CV cutoff of 30% or even 20% (Fig. 7) [10] results in no further filtering of the remaining low quality features in the test set. With all steps of the data-adaptive pipeline, the CRC dataset was reduced to just 3,009 features. The data-adaptive filtering removed 76% of features identified as low quality and retained 72% of those identified as high quality in the test set (Fig. 8).

When the data-adaptive pipeline was applied to the publicly available urine dataset [19], 83% of the high quality features in the test set were retained and 74% of the low quality features in the test set were removed. We used a percent missing cutoff of 69% (median of percent missing in the low quality feature training set) and an ICC cutoff of 0.35 (lower whisker of the box plot of ICC values for low quality features in the training set). When the data-adaptive pipeline was applied to the public cell line dataset [20], 79% of the high quality features in the test set were retained and 76% of the low quality features in the test set were removed. We used a percent missing cutoff of 27% (median of percent missing values in the low quality feature training set) and an ICC cutoff of 3.8×10^−9^ (median of ICC values for low quality features in the training set).

## Discussion

We recognize that our data-adaptive pipeline involves several steps of manual work, such as the visual identification of high and low quality features and the selection of filtering cutoffs. Such methods do present the opportunity for user error, but we argue that such error will not effect the end results of a study. To our knowledge, *xcms* does not provide peak quality scores for an automated identification of high and low quality peaks. Furthermore, as stated previously, in the vast majority of cases the contrast between images of high and low quality features is striking. Occasional miss-classification of features as high or low quality will not considerably affect the distributions of the feature characteristics used to select the cutoffs, and therefore will not have a large impact on final filtering results. We see the manual selection of filtering cutoffs based on thorough data visualization as an advantage of our proposed pipeline. Researchers may likely have specific requirements for the balance between removing low quality and retaining high quality features depending on their scientific question of interest, their analysis plan or the size of their data. Manual selection of filtering cutoffs, as opposed to using pre-determined cutoffs, allows researchers to adjust the stringency of their feature filtering to fit the needs of their study.

## Conclusions

Pipelines such as *Workflow4Metabolomics* and *MetaboAnalyst* have been crucial for advancing LC-MS based untargeted metabolomics. The aim of our work is to assist users in applying appropriate filtering methods for their specific data instead of relying on default, non-specific filtering parameters. Given the inherent heterogeneity of metabolomic studies, we argue that feature filtering should be data-adaptive. Here, we provide filtering criteria for each step in a metabolomic pipeline and discuss how to choose cutoffs based on data visualization and distributional properties of high and low quality features. Because of the random noise present in untargeted LC-MS data, we also encourage investigators to visually inspect features of interest for peak morphology and integration prior to including them in analyses of biological variability. We appreciate that our data-adaptive filtering method requires more effort than selecting default or common cutoffs, but argue that the improved data quality will greatly improve statistical analyses performed in applications involving biomarker discovery and pathway characterization leading to more robust and reproducible findings.

## Additional file

Additional file 1 Filtering procedures for untargeted LC-MS metabolomics data. This file illustrates the application of the data-adaptive filtering pipeline to an additional dataset generated in our laboratory. (PDF 379 kb)

## Abbreviations

CRC: Colorectal cancer
CV: Coefficient of variation
EIC: Extracted ion chromatogram
ICC: Intra-class correlation coefficient
LC-MS: Liquid chromatography mass spectrometry
MD-plot: Mean-difference plot
QC: Quality control
RSD: Relative standard deviation
