# Supplementary material for: Filtering procedures for untargeted LC-MS metabolomics data
Source: BMC Bioinformatics. 2019 Jun 14;20:334. doi: 10.1186/s12859-019-2871-9 (PMC6570933; doi:10.1186/s12859-019-2871-9)
Supplement: Supplementary file 1 — Filtering procedures for untargeted LC-MS metabolomics data. This file illustrates the application of the data-adaptive filtering pipeline to an additional dataset generated in our laboratory. (PDF 379 kb) [file 12859_2019_2871_MOESM1_ESM.pdf]

METHODOLOGY

Additional File 1: Filtering procedures for untargeted LC-MS metabolomics data.

Courtney Schiffman, Lauren Petrick, Kelsi Perttula, Yukiko Yano, Henrik Carlsson, Todd Whitehead, Catherine Metayer, Josie Hayes, Stephen Rappaport and Sandrine Dudoit

Full list of author information is available at the end of the article

Additional File 1 contains plots and results of the data-adaptive filtering method applied to a second dataset under a different analytical platform and biological matrix generated from our lab. This second dataset is an untargeted LC-HRMS metabolomics dataset generated on a platform consisting of an Agilent 1290 UPLC coupled to an Agilent 6550 QToF MS. The dataset represents the metabolomes of 4.7-mm punches from archived neonatal blood spots (NBS) of 309 incident case subjects that were obtained for the California Childhood Leukemia Study [1], as described in [2]. Over 60,000 features were initially measured in each of 309 NBS samples that were analyzed in four batches (along with blank and QC samples). We note that, unlike the 3 datasets presented in the main text, features are not split into a training and testing set for this demonstration of the filtering pipeline.

MD-plot Filtering

As with the CRC dataset presented in the main text, four clusters of features can be clearly identified in the MD-plot (Fig. 1). Again, the four clusters correspond to the number of blank samples the features are detected in ($0, 1, 2$ or 3 blank samples). Unlike the CRC dataset, high quality features can be found in each of the four clusters (Fig. 1), and filtering is performed for each cluster accordingly. For the cluster corresponding to features detected in all three blank samples (Fig. 1 (b)), the absolute value (green lines) of the lower quartile (purple lines) of differences below the zero-difference line is used as a filtering cutoff. This cutoff is selected because it does not remove any high quality features but removes a considerable number of low quality ones. For the remaining three clusters (Fig. 1 (c) and (d)), lower percentiles (i.e. more stringent cutoffs) are selected as cutoffs based on the position of the high quality features within the clusters. This filtering is performed for each of the four batches present in the experiment, and taking the intersection of the resulting features removes 80% of the total features, leaving 12,371 features.

Percent Missing Filtering

A difference in distribution of percent missing values between the remaining high and low quality features can again be seen for the NBS dataset (Fig. 2). For example, most of the high quality features have percent missing values below 28% in the first batch. This distribution visualization and filtering is repeated for the remaining three batches. Using p-values from the Fisher exact test (the phenotype of interest

in this dataset concerns a specific sub-type of Leukemia), 124 features are retained regardless of their percent missing values because their p-values are below the one hundredth percentile of the p-value distribution, 0.025. Taking the intersection of the remaining features in each batch after percent missing filtering and keeping features with Fisher exact test p-values smaller than the threshold removes another 79% of the features, resulting in 2,642 features.

ICC Filtering

As with the CRC dataset, the remaining high quality features tend to have higher ICC values than the low quality ones (Fig. 3). In the first batch, we remove features with estimated ICC values less than 0.72. Similar visualization and filtering is done for the remaining three batches. Taking the intersection of the remaining features results in 778 features. Again, traditional CV cutoffs between 20 and 30% remove at most one poor quality feature in each batch.

Author details

[1]Division of Biostatistics, UC Berkeley, 94720 Berkeley, US. [2]Department of Environmental Medicine and Public Health, Icahn School of Medicine at Mount Sinai, New York, US. [3]Department of Statistics, UC Berkeley, 94720 Berkeley, US. [4]Division of Environmental Health Sciences, UC Berkeley, 94720 Berkeley, US. [5]Division of Epidemiology, UC Berkeley, 94720 Berkeley, US. [6]Center for Integrative Research on Childhood Leukemia and the Environment, UC Berkeley, 94720 Berkeley, US.

References

1. Metayer, C., Zhang, L., Wiemels, J., Bartley, K., Schiffman, J., et al.: Tobacco smoke exposure and the risk of childhood acute lymphoblastic and myeloid leukemias by cytogenetic subtype. Cancer Epidemiol Biomarkers Prev **22**(9) (2013)
2. Petrick, L., Edmands, W., Schiffman, C., Grigoryan, H., Perttula, K., et al.: An untargeted metabolomics method for archived newborn dried blood spots in epidemiological studies. Metabolomics **13**(27) (2017)
3. Giacomoni, F., Corguille, G.L., Monsoor, M., Landi, M., Pericard, P., et al.: Workflow4metabolomics: A collaborative research infrastructure for computational metabolomics. Bioinformatics (2014)

Figures

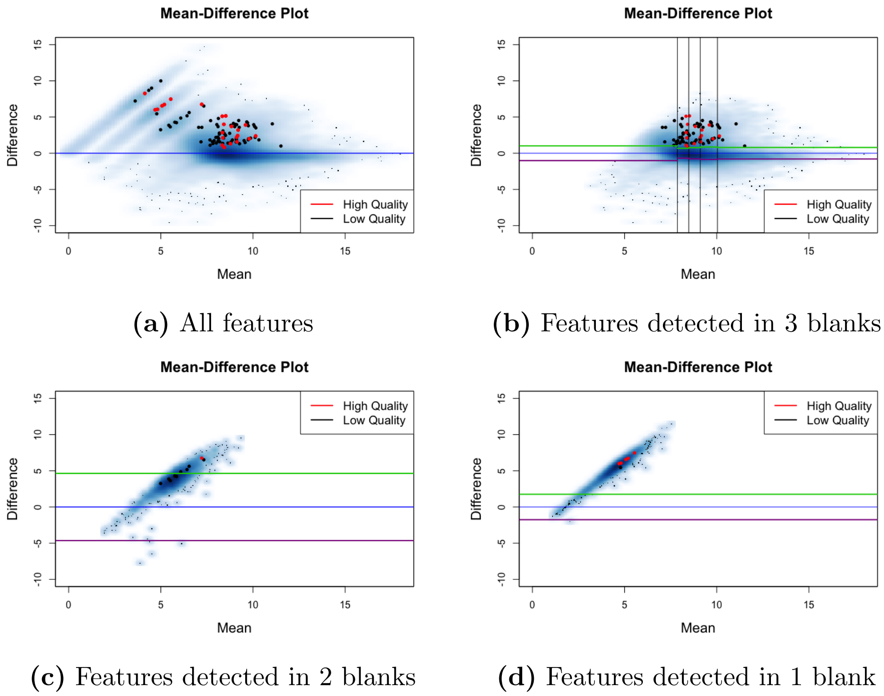

(a) All features

(b) Features detected in 3 blanks

(c) Features detected in 2 blanks

(d) Features detected in 1 blank

Figure 1 MD-plot for the NBS dataset, batch 1 (a) An MD-plot for all features from the NBS dataset. The four identifiable clusters correspond to features found in 0, 1, 2, or 3 of the blank samples. The x-axis represents the average of the mean log abundances in blank and biological samples, whereas the y-axis represents the difference in mean log abundances between biological and blank samples. (b) Cluster from (a) corresponding to features detected in all 3 blank samples. The purple line indicates the lower-quartile of abundances below the zero difference line for each of the five partitions. Features above the green line (absolute value of the purple line) are retained. (c) Cluster from (a) corresponding to features detected in 2 blank samples. The purple line indicates the first percentile of abundances below the zero difference line. Features above the green line (absolute value of the purple line) are retained. (d) Same as (c) but for features detected in 1 blank samples, and using the fifth percentile.

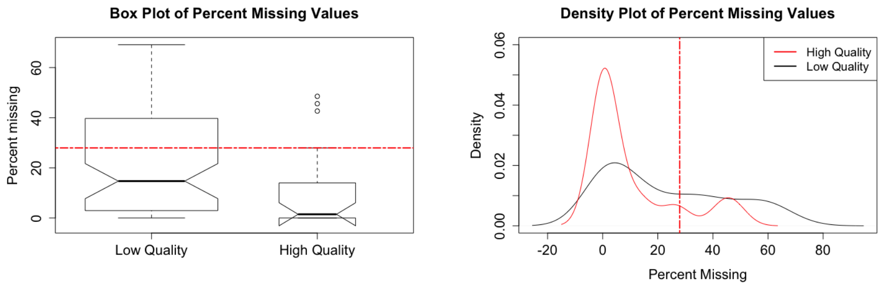

Figure 2 Filtering by Percent Missing for the NBS dataset, batch 1. In each of the four batches, high quality features tend to have fewer percent missing values than the low quality features. For batch one, we choose a cutoff of 27%, the upper whisker of the boxplot corresponding to the high quality features. This cutoff only removes a few of the high quality features while removing 40% of the low quality ones.

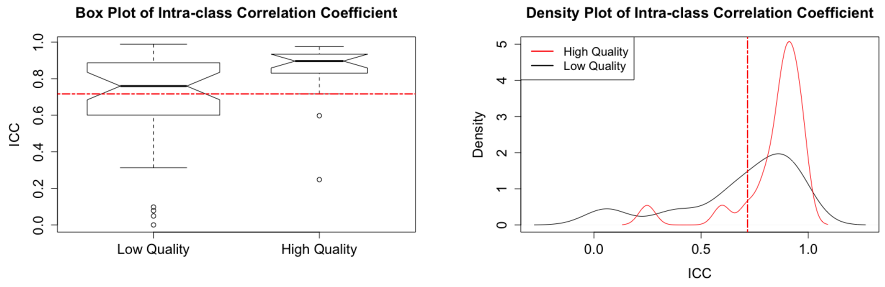

Figure 3 Filtering by Intra-class Correlation Coefficient for the NBS dataset, batch 1. In each of the four batches, high quality features tend to have higher estimated ICC values than the low quality features. For batch one, we choose a cutoff of 0.72, the lower whisker of the boxplot corresponding to the high quality features, which can also be seen to fall just to the left of the mode of the high quality feature distribution. This cutoff only removes a few of the high quality features while removing almost 50% of the remaining low quality ones.

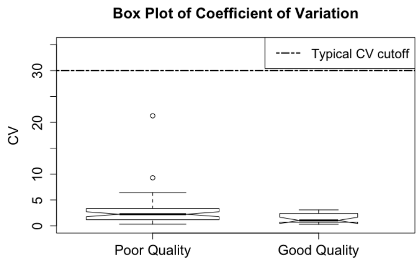

Figure 4 Filtering by Coefficient of Variation for the NBS dataset, batch 1. A typical CV filtering cutoff is 30% (horizontal black line) [3]. For the NBS dataset, this cutoff does not remove any of the low quality features.
